# An electrochemical aptasensor for methylamphetamine rapid detection by single-on mode based on competition with complementary DNA

**DOI:** 10.1038/s41598-024-59505-6

**Published:** 2024-04-23

**Authors:** Wenzhuo Chang, Zhixiang Zheng, Yongjun Ma, Yongling Du, Xuezhao Shi, Chunming Wang

**Affiliations:** 1 Key Laboratory of Evidence Science Techniques Research and Application of Gansu Province, Gansu University of Political Science and Law, Lanzhou, 730070 China; 2 College of Chemistry and Chemical Engineering, Northwest Normal University, Lanzhou, 730070 China; 3 College of Chemistry and Chemical Engineering, Lanzhou University, Lanzhou, 730000 China

**Keywords:** Methylamphetamine (MAMP), Point-of-care testing (POCT), Aptamer, Competitive, Complementary DNA, Electrocatalysis, Analytical chemistry, Bioanalytical chemistry

## Abstract

A simple and rapid electrochemical sensing method with high sensitivity and specificity of aptamers was developed for the detection of methylamphetamine (MAMP). A short anti-MAMP thiolated aptamer (Apt) with a methylene blue (MB) probe at 3ʹ-end was immobilized on the surface of a gold electrode (MB-Apt-S/GE). The electrochemical signal appeared when MAMP presenting in the sample solution competed with cDNA for binding with MB-Apt-S. Under optimized conditions, the liner range of this signal-on electrochemical aptasensor for the detection of MAMP achieved from 1.0 to 10.0 nmol/L and 10.0–400 nmol/L. LOD 0.88 nmol/L were obtained. Satisfactory spiked recoveries of saliva and urine were also obtained. In this method, only 5 min were needed to incubate before the square wave voltammetry (SWV) analysis, which was much more rapid than other electrochemical sensors, leading to a bright and broad prospect for the detection of MAMP in biological sample. This method can be used for on-site rapid detection on special occasions, such as drug driving scenes, entertainment venues suspected of drug use, etc.

## Introduction

According to the world drug report 2022, released by the United Nations Office on Drugs and Crime (UNODC), June 24, 2021, an estimated 275 million people worldwide will have used drugs in 2021. The number of drug users has increased by 22 per cent since 2010 and is expected to continue to increase by 11 per cent by 2020. The report also states that in 2019, drug use caused nearly half a million deaths, more than 36 million drug-related mental disorders, and mental disorders caused by heavy drug use kill 18 million people. And the largest number of amphetamine-type stimulants seized in 2019 was methamphetamine. Between 2015 and 2019, the number of most drugs worldwide increased the most with drugs of plant origin, followed by opioids and amphetamine-type drugs (most notably methamphetamine).

Known as the second most widely used illicit drug in the worldwide after cannabis, methylamphetamine (MAMP) is an addictive central nervous stimulant^1,2^. By blocking serotonin, dopamine and norepinephrine receptors in the brain can result in irreparable problems^2,3^. Meanwhile, the increased heart rate, blood pressure and respiratory problems in MAMP consumers may lead to stroke and death^4,5^.

Therefore, it is urgent to strengthen the monitoring of the scale of drug abuse and assess the extent of harm to society. Because of the timeliness of on-site investigation, it is urgent to develop direct and rapid detection technology. Among many detection technologies, the combination of electrochemical technology and new materials has become a possibility to break through this bottleneck. Thus, it is very important to develop a reliable method to monitor the presence of drugs in human body fluids.

The literature showed that several methods have been developed to detect MAMP, such as gas chromatography (GC)^6^, high-performance liquid chromatography (HPLC)^7^, GC-mass spectrometry (GC–MS)^8,9^ and electrochemiluminescence (ECL)^10^. These methods could supply accurate results of MAMP in complex samples including human urine and blood. However, they have several drawbacks, such as expensive instruments, complex sample preparation and not suitable for real-time detection^11–15^. Therefore, it’s very necessary to set up a simple, accurate and fast method for the detection of MAMP. Electrochemical sensors is a simple and inexpensive method, which is also attractive because of its sensitive continuous real-time measurements and reducing analysis time compared with the methods described above^16,17^.

Nowadays, electrochemical sensors based on aptamer have attracted increasing attentions due to its advantages, such as low cost, high stability, small size, easy synthesis and high sensitivity^18–21^. Combined with aptamer strategies, the biosensors have already been used to the detection of MAMP based on the specific binding ability between aptamer and MAMP^22–26^. However, traditional aptamer sensing methods have not been used widely because of low efficiency and sensitivity due to tedious and time-consuming detection steps and long sample incubation time. Additionally, some MAMP electrochemical aptasensors cannot be reused. Therefore, it is urgent to develop a new aptasensor which could satisfy the requirement for efficiency detection.

In this work, we reported a simple electrochemical aptasensor for rapid detection of MAMP. This strategy used an anti-meth thiolated aptamer (Apt), in which a methylene blue (MB) modified on the 3’-end, as an electrochemical aptasensor (MB-Apt-S). MB-Apt-S was anchored on the surface of the gold electrode by Au–S bond. The complementary DNA strand (cDNA) of MB-Apt-S could hybridize with MB-Apt-S to form a double-helix structure in the absence of MAMP, making MB far from the electrode surface and resulting in low current. While in the presence of MAMP, it could compete with cDNA for binding to MB-Apt-S resulted in a hairpin structure, in which the MB was close to the electrode surface leading to the increase of response current.

Under optimized experimental conditions, the liner range of MAMP from 1.0 to 10.0 nmol/L and 10.0–400 nmol/L with the limit of detection (LOD, S/N = 3) 0.88 nmol/L were obtained. The detection procedure was simple and fast and only about 5 min were needed to incubation with sample before electrochemical scanning. Moreover, this sensor can be reused because of its stability and reproducibility. Consequently, it is a very promising sensor for detection of MAMP in real sample applications.

## Experimental

### Chemicals and reagents

One short anti-MAMP aptamer (5ʹ-ACGGTTGCAAGTGGGACTCTGGTACCGT-3’) was modified with MB at 3ʹ-end and thiolated at the 5′-end, named as MB-Apt-S^27^. All standard MB-Apt-S and its cDNA were commercially supplied by Sangon Biotech Co., Ltd (Shanghai, China) and purified by HPLC. The detailed sequences were listed in Table 1. Tris (2-carboxyethyl) phosphine hydrochloride (TCEP), 6-mercapto-1-hexanol (MCH) and methylene blue (MB) were obtained from Macklin Co., Ltd (Shanghai, China). The deoxyephedrine and methylamphetamine solution was obtained from Shanghai Academy of Criminal Science and Technology (Shanghai, China). Double-distilled water was used to prepare all the solutions. The 1 × PBS buffer (pH = 7.4) contained 137 mmol/L NaCl, 2.7 mmol/L KCl, 10 mmol/L Na_2_HPO_4_ and 2 mmol/L KH_2_PO_4_ and the human urine samples were obtained from healthy laboratory experimenters. Prior to the electrochemical measurements, the fresh urine was filtered through a membrane of 0.45 μm diameter and diluted for 10 times using 1 × PBS buffer (pH = 7.4).

**Table 1:** DNA oligonucleotides.

Name	Sequence (5ʹ–3ʹ)
MB-Apt-S	HS-SH-(CH_2_)_6_-ACGGTTGCAAGTGGGACTCTGGTACCGT-Methylene Blue
cDNA(C8)	GCAACCGT
cDNA(C10)	TTGCAACCGT
cDNA(C12)	ACTTGCAACCGT
cDNA(C14)	CCACTTGCAACCGT
cDNA(C16)	TCCCACTTGCAACCGT

The electrochemical measurement was performed on the electrochemical workstation (CHI660E, CH Instrument Co., Shanghai). The electrochemical experiments were conducted via traditional 3-electrode system with a modified gold electrode (Φ = 2.0 mm) as a working electrode, a saturated Ag/AgCl (1 mol/L KCl solution) as the reference electrode, and a Pt wire as an auxiliary electrode.

### Fabrication of the aptamer modified electrode

The MB-Apt-S modified electrode was prepared according to literatures^24,28^. The GE was first polished to a mirror finish using 0.05 μm aluminum oxide powders on a microcloth, and then ultrasonically washed by double-distilled water, ethanol and double-distilled water. Subsequently, the gold electrode was cleaned by electrochemical method according to the previous report^28^. The gold electrode (GE) was electrochemically cleaned in 0.5 M H_2_SO_4_ aqueous solution (3 mL) by an oxidation step (2 V for 5 s) and then a reduction step (-0.35 V for 10 s), before cyclic voltammetry (CV) in the range of -0.35 V ~ 1.55 V (20 scans at a scan rate of 4 V/s, followed by 10 scans at a scan rate of 0.1 V/s). After that, a second electrochemical cleaning was performed in a solution of 0.1 M sulfuric acid containing 0.01 M potassium chloride. In details, a set of CV scans were performed in four different potential ranges: (a) potential range from 0.2 to 0.75 V; (b) potential range from 0.2 to 1 V; (c) potential range from 0.2 to 1.25 V; (d) potential range from 0.2 to 1.5 V. 10 segments for each potential range, at a scan rate of 0.1 V/s. Finally, the gold electrode was rinsed with ultrapure water and blow-dried by nitrogen, and it was ready for modification.

Before immobilizing MB-Apt-S, the thiolated MB-Apt-S was mixed with fresh TCEP (2 mmol/L) and left at 4 ℃ for 1 h to reduce the disulfide bond (S–S bond) to activate MB-Apt-S. The activated MB-Apt-S stock solution was then diluted by PBS buffer (1 × PBS, pH = 7.5). Subsequently, 50 μL of the activated MB-Apt-S solution was added dropwise to the surface of the gold electrode and incubated at room temperature for 1 h. Ultimately, the MB-Apt-S was immobilized on the surface of the gold electrode through Au–S and we obtain the modified electrode. After that, the electrode was incubated in ethanol solution with 1 mmol/L MCH at room temperature for 2 h. The thiol molecules in the MCH solution could occupy the active sites on the GE surface to passivate the GE, which was conducive to the formation of a well-aligned DNA monolayer^29^. After a thorough wash, the modified electrode (MB-Apt-S/GE) was finally obtained.

### MAMP detection

The MB-Apt-S/GE was incubated at 4 °C in 1 × PBS (pH = 7.5) buffer (50 μL) containing MAMP and cDNA for 5 min. The electrode was washed well with double-distilled water and subjected to square wave voltammetry (SWV) analysis in 5 mL of 1 × PBS (pH = 7.5) solution. The peak current for methylene blue (MB) was approximately at − 0.25 V. The parameters were set as follows, the scan range from 0 to − 0.5 V, the frequency was 60 Hz and an amplitude of 25 mV. After each measurement, the electrode was reactivated by immersing in 5 mol/L NaCl solution for 20 min, followed by washing with double-distilled water for 3 min to remove the cDNA and MAMP that bounded to the electrode surface. Specific experimental data are available in the Supporting Information.

## Results and discussion

### Principle of the electrochemical aptasensor for MAMP

**Scheme 1:**
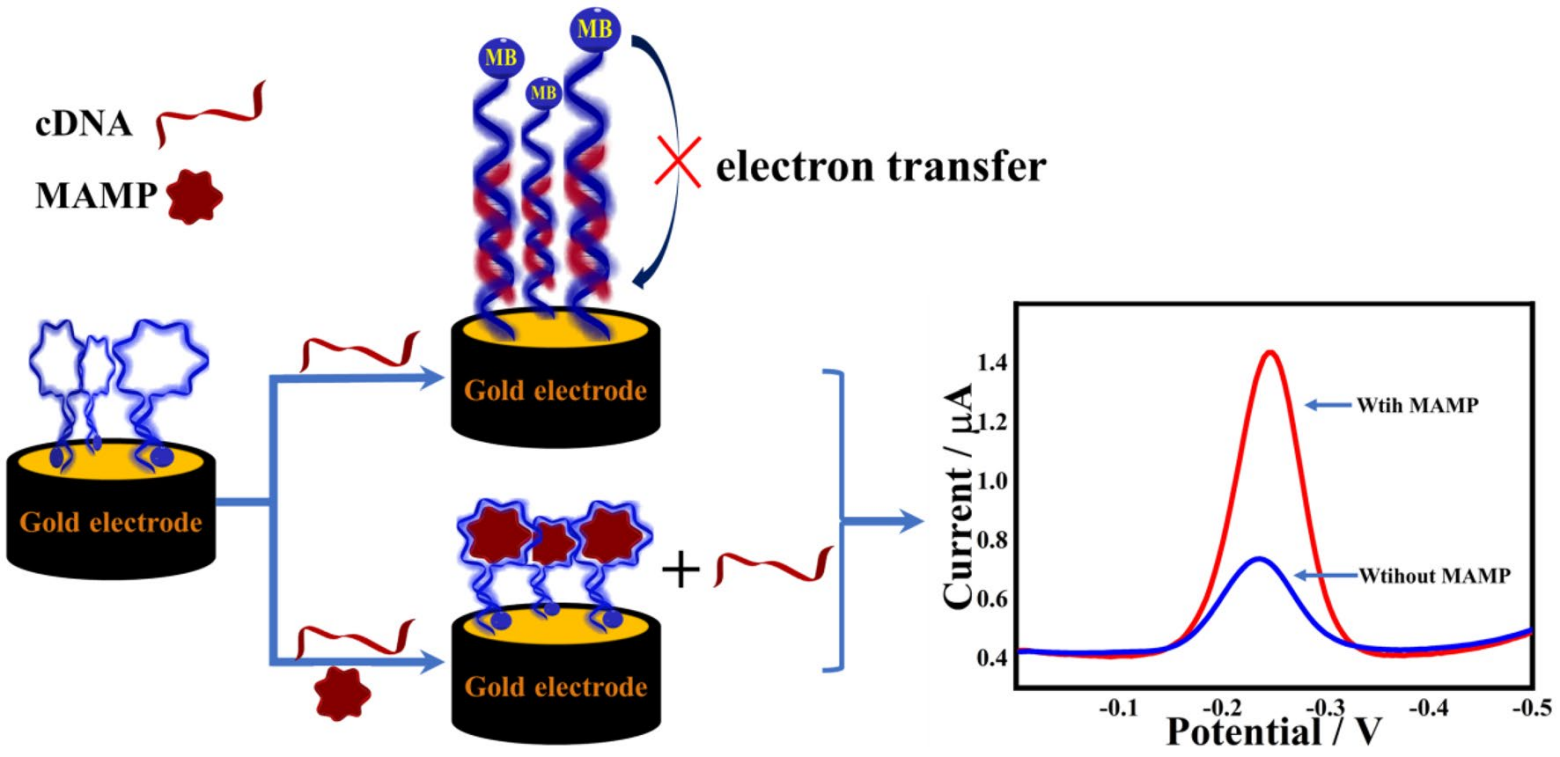
Schematic diagram of the signal-on electrochemical aptasensor for MAMP detection by using a gold electrode modified with aptamer having MB label at the terminal and a cDNA. Detection of MAMP is achieved by measuring the change of current of MB label.

In this experiment, a competitive binding strategy between cDNA and MAMP was used for the quantitative analysis of MAMP. In a previously reported study^30^ in which the amount of bound aptamer to the MAMP modified matrix was measured, MAMP aptamer has been shown to display a Kd value of ∼ 100 nM. Due to limited laboratory conditions, the Kd values of the complementary chains with MB-Apt-S were not measured. Through the optimisation of conditions and experimental phenomena, we can conclude that MB-Apt-S binds stronger to MAMP than cDNA. After MB-modified Apt was immobilized on the gold electrode surface by Au–S bonding, the addition of MCH not only passivated the electrode by binding the blank active site, but also kept the Apt in an upright position under the effect of spatial resistance, which resulted in an aptamer-sensing interface with specific recognition ability. Before the incubation, the modified electrode (MB-Apt-S/GE) was scanned using SWV to obtain the corresponding peak current value, noted as i_p initial_. In the absence of the MAMP, the MB-Apt-S/GE was incubated with a buffer containing cDNA, which complementarily bound to the surface aptamer to form a rigid DNA double-stranded structure, keeping the MB away from the gold electrode surface. When MAMP and cDNA were present at the same time, they will compete for the binding of MB-Apt-S. As MB-Apt-S had strong affinity with MAMP, the specific recognition will form a stable complex and produce a hairpin structure. At this time, the distance between MB and gold electrode surface decreased so that the electron collision transfer rate accelerated. A larger electrochemical signal was generated and the current value was recorded as i_p MAMP_. The difference of SWV peak current before and after adding MAMP was used as the response current to achieve the electrochemical detection of MAMP (Scheme 1). Apt and cDNA were designed according to previous reports^24^. A 28-base specific recognition aptamer was used as the sensing element, which was obtained by truncating a long specific sequence recognition aptamer of MAMP.

### Characterization of the electrochemical aptasensor

**Figure 1:**
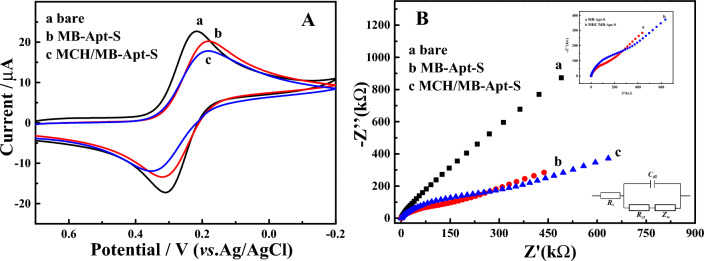
Cyclic voltammetry response (**A**) and electrochemical impedance spectroscopy (**B**) of bare GE (a), MB-Apt-S/GE (b) and passivated MB-Apt-S/GE (c). All measurements were carried out in a buffer solution, 10 mM Tris–HCl (pH 7.5) containing 0.1 mol/L KCl and 5 mM [Fe(CN)_6_]^4−//3−^. CV was conducted in the potential range from -0.2 V to 0.6 V at a scan rate of 50 mV/s. Equivalent circuit corresponding to the EIS spectrum was shown. R_s_ and R_et_ were electrolyte and electron transfer resistances, Z_w_ was the Warburg impedance, and C was the capacitance of the electrode surface/solution interface.

The fabrication procedure of the aptasensor was monitored by measurements of cyclic voltammetry (CV) and electrochemical impedance spectroscopy (EIS), respectively. In this part, the electrolyte solution was 10 mmol/L Tris–HCl buffer (pH 7.5) containing 0.1 mol/L KCl and 5 mmol/L K_3_[Fe(CN)_6_]/K_4_[Fe(CN)_6_] (1:1). CV measurements were conducted in the range from − 0.2 to 0.6 V at a scan rate of 50 mV/s. EIS measurements were performed in a frequency range of 0.1 Hz to 10 kHz with an amplitude of 250 mV. As shown in Fig. 1A, a pair of distinct redox peaks for [Fe(CN)_6_]^3−/4−^ can be seen on the bare GE (curve a). The conductivity of the GE was decreased when MB-Apt-S incubated on the bare electrode (curve b), which should be caused by the poor conductivity of MB-Apt-S, indicating that MB-Apt-S was successfully modified on the GE surface. After passivating with MCH, the peak current decreased again (curve c) because of the MCH was successfully modified onto gold surface. The electrode preparation process was further verified by EIS, which usually consists of a half circle in the high frequency region and a straight line in the low region. The semicircles reflect the electron transfer process and the straight lines represent the diffusion process. As shown in Fig. 1B, the diameter of the semicircle of MB-Apt-S/GE (curve b) was larger than that of the bare electrode (curve a), confirming that the MB-Apt-S/GE electron transfer process had a larger resistance. From the inset of Fig. 1B, it can be seen that the semicircle diameter was larger when MB-Apt-S/GE was passivated by MCH, indicating an increased resistance to charge transfer. These results proved that the modified electrode was successfully prepared by the layer-by-layer modification method.

### Feasibility validation experiments of modified electrode sensing

**Figure 2:**
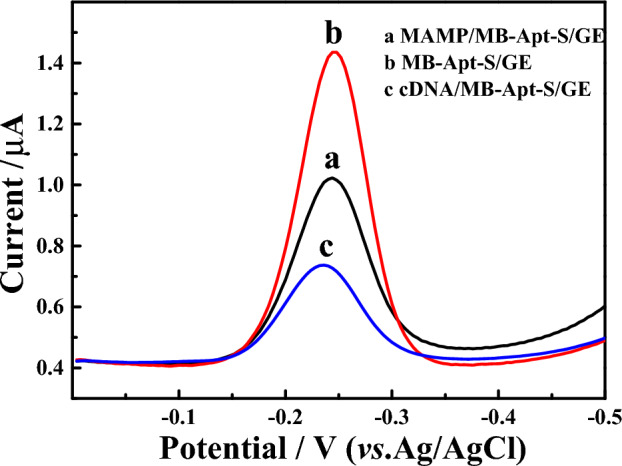
SWV peak currents of MB-Apt-S/GE were recoded (a) after incubation with sample containing cDNA (200 nmol/L) and MAMP (200 nmol/L); (b) before incubation of sample solution; (c) after incubation with sample containing cDNA (200 nmo/L).

To verify the feasibility of the modified electrode, MB-Apt-S/GE was incubated in different buffers. The peak current values of MB were measured using SWV. As shown in Fig. 2, the current response (i_p blank_, 3.291 × 10^−7^A) (curve c) of the electrode incubated with the cDNA-only buffer solution was significantly lower than that without incubation (curve b), indicating that the complementary binding of MB-Apt-S to cDNA kept the MB marker away from the electrode surface and reduced the peak current. When 200 nmol/L MAMP was added to the cDNA solution, the peak signal current (i_p MAMP_, 6.028 × 10^−7^A) (curve a) was higher than that of i_p blank_, indicating that the binding of the aptamer to the target occurred.

After the assay, the electrode was immersed in 5 mol/L NaCl solution for 20 min and then washed with double-distilled water for 3 min, in which case the affinity of the aptamer decreased significantly and the hydrogen bonding force was weakened^31,32^, and the cDNA and MAMP would be released. Although the current signal will be reduced at this point compared to the newly modified MB-Apt-S/GE, the modified electrode will still have a good signal response to MAMP and the modified electrode can be reused.

### Optimization of experimental conditions

**Figure 3:**
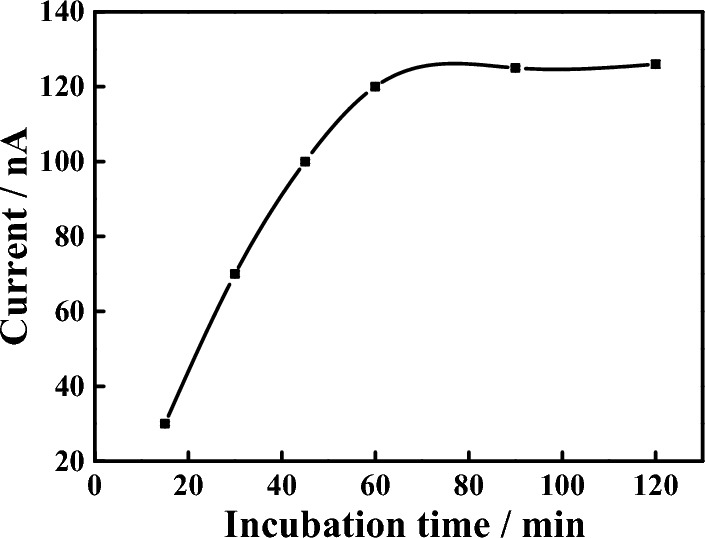
MB-Apt-S incubation time optimisation curve.

In order to improve the performance of the sensor, the experimental conditions were optimized. Firstly, we optimised incubation time for MB-Apt-S. Incubation too long will reduce detection efficiency. On the contrary, MB-Apt-S may bind with electrode inadequately. As shown in Fig. 3 , 1 h was the most appropriate incubation time according to our test.

**Figure 4:**
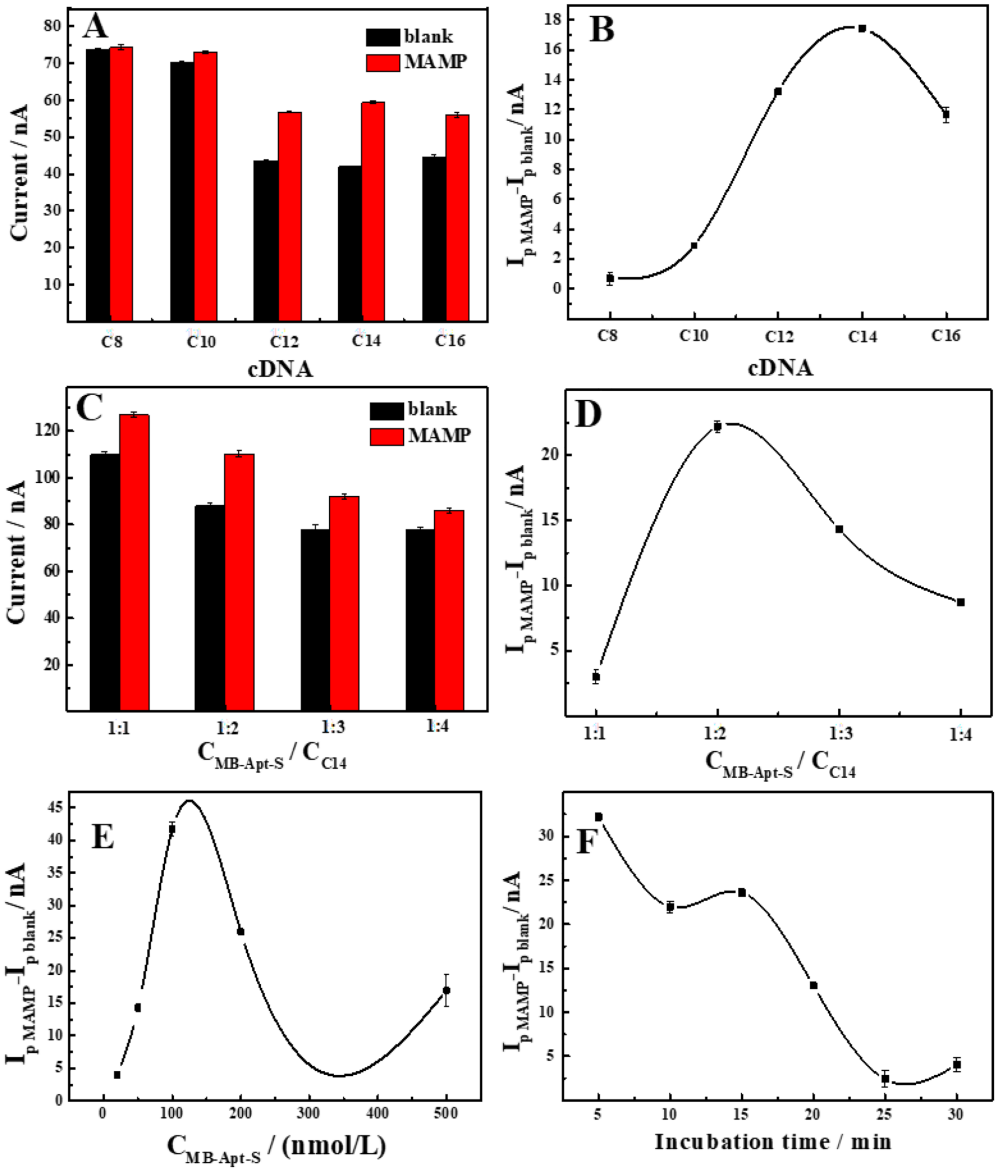
(**A**) Peak currents of MB-Apt-S/GE before and after incubation with 200 nM MAMP in different lengths of cDNA. (**B**) Effects of lengths of cDNA on signal change (i_p MAMP_ − i_p blank_) caused by MAMP. (**C**) Peak current values before and after incubation of 200 nmol/L MB-Apt-S with different ratios of cDNA and 200 nmol/L MAMP. (**D**) change in peak current values due to incubation with different ratios of cDNA. (**E**) Effect of MB-Apt-S modifier concentration on 200 nmol/L MAMP peak current variation. (**F**) Effect of 200 nmol/L MAMP incubation time on MAMP peak current variation.

Then, the cDNA length was optimized and shown in Fig. 4A. i_p blank_ decreased with increasing length of incubated cDNA, which was due to the fact that the longer the cDNA length, the stronger the complementary binding, and when the length was more than 12 bases, the current value tended to stabilize and does not decrease further with increasing cDNA length. Under all conditions, the addition of MAMP caused an increase in the peak current i_p MAMP_, however, when the cDNA was very long, the aptamer bound too strong for MAMP to displace the cDNA, so i_p MAMP_ tended to decrease. As shown in Fig. 4B, MAMP addition will cause a change in the MB current response value, with the largest change in current (i_p MAMP_—i_p blank_) when measured using C14, and therefore C14 was chosen as the length-optimal complementary strand.

The effect of the MB-Apt-S to cDNA concentration ratio was also examined. In order to gain the biggest current change value, the cDNA concentration should be excessive and the MB-Apt-S to cDNA ratios of 1:1, 1:2, 1:3 and 1:4 was measured for comparison. As shown in Fig. 4C, the i_p blank_ decreases due to an increase in the complementary strand concentration. As can be seen in Fig. 4D, the MB-Apt-S /cDNA ratio of 1:2 was chosen as the optimal condition for subsequent experiments.

Then, the effect of the concentration of the MB-Apt-S modification solution was investigated. As the concentration of the MB-Apt-S solution increased, the peak current value of the modified electrode in the blank solution will keep increasing. But when the MB-Apt-S on the electrode were too crowded, the MB will be repelled away from the electrode surface, which will lead to a decrease in the signal response. As shown in Fig. 4E, the electrochemical response of 200 nmol/L MAMP was measured at different concentrations of the MB-Apt-S modification solutions ranging from 10.0 to 500 nmol/L. The current signal values reached the highest when the concentration was 100 nmol/L. Therefore, 100 nmol/L was chosen as the best MB-Apt-S modification solution’s concentration (Supplementary Information).

Finally, the effect of the MAMP incubation time was optimized. As shown in Fig. 4F, the MAMP current responses gradually diminished with increasing of incubation time. The results showed that the binding ability of MB-Apt-S to cDNA increased with increasing incubation time, which was not conducive to the competitive binding reaction of MAMP. The longer the aptamer react with the cDNA, the more base-pair were recognized and therefore their binding became stronger. And after completely binding, it will be more difficult for MAMP to competitively bind to the cDNA. The optimal incubation time 5 min was chosen. Meanwhile, the effects of the modified electrode on the current response values of MAMP were compared at room temperature and 4 ℃ incubation temperature. The results showed that the low temperature was favorable for the binding of MAMP to MB-Apt-S. This is consistent with the literature^33^.

### Effect of CV scan rate

**Figure 5:**
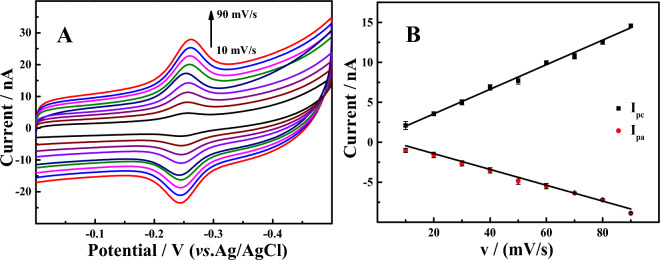
(**A**) CV curves of MB-Apt-S/GE at different scan rates (10, 20, 30, 40, 50, 60, 70, 80 and 90 mV/s) in blank buffer solution. (**B**) The relationship between the peak current of MB and the scan rate.

In order to obtain kinetic information of the modified electrode, the effect of scan rate on the electrode surface was investigated through CV. MB-Apt-S/GE was transferred to blank buffer (1 × PBS) for scanning. As shown in Fig. 5, the redox peak currents increased with increasing scan rate and were proportional to the scan rate, and the corresponding linear equations were as follows:

$$ \begin{aligned} & {\text{I}}_{{{\text{pc}}}} \left( {\mu {\text{A}}} \right) = 0.{5}0{83} + 0.{1521}v\left( {{\text{mV}}/{\text{s}}} \right),({\text{R}}^{{2}} = 0.{9944}) \\ & {\text{I}}_{{{\text{pa}}}} \left( {\mu {\text{A}}} \right) = 0.{2}0{28} - 0.0{9631}v\left( {{\text{mV}}/{\text{s}}} \right),({\text{R}}^{{2}} = 0.{9959}) \\ \end{aligned} $$

The results indicated that the electrochemical process of MB-Apt-S on the electrode surface was an adsorption-controlled process, which was consistent with the typical behavior of the substance immobilized modification^34^.

### Detection of MAMP

**Figure 6:**
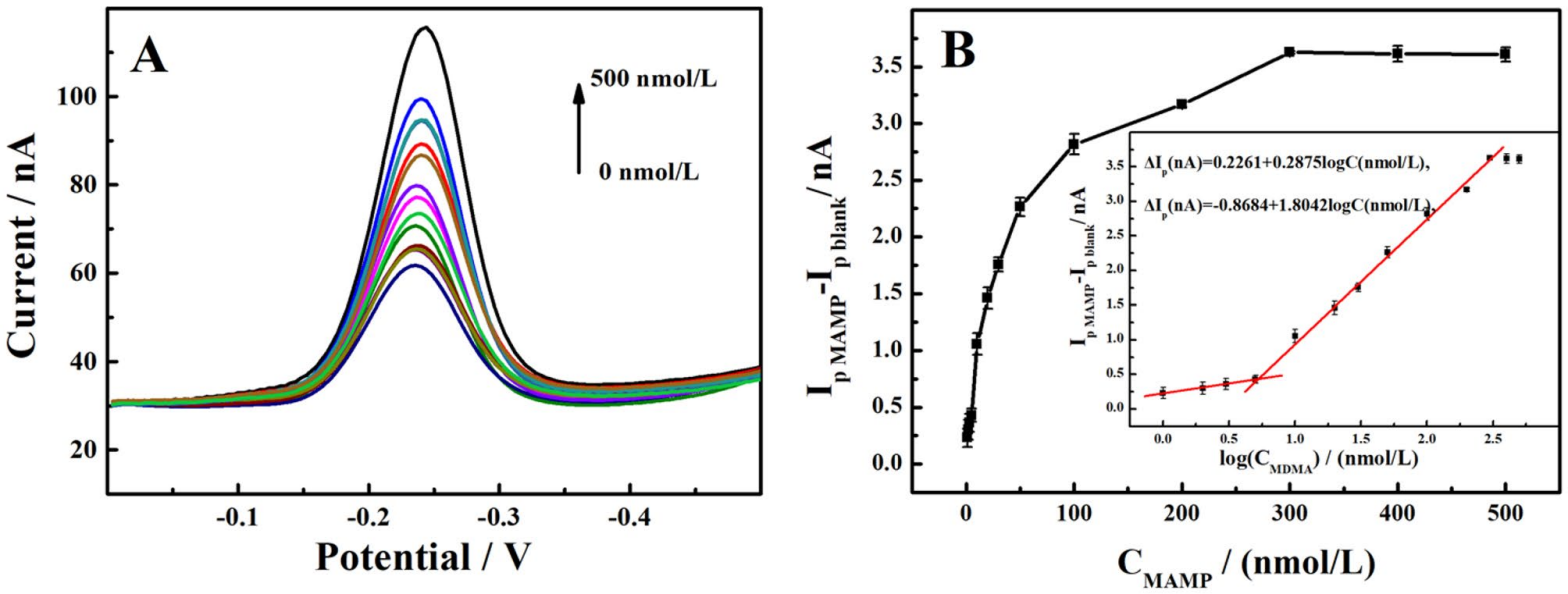
(**A**) SWV curves of 0 to 500 nmol/L MAMP on MB-Apt-S/GE. (**B**) MB peak current increment versus MAMP concentration (inset shows calibration curve of MB peak current increment versus log c_MAMP_).

The effect of the MAMP concentration on MB-Apt-S/GE was investigated under optimal experiment conditions to study the analytical detection performance of this modified electrode for MAMP. As shown in Fig. 6, the incremental values of MAMP peak currents on MB-Apt-S/GE were linearly related to the logarithmic values of its concentration in the range of 10.0–400 nmol/L, and the regression equation was:

$$ \Delta {\text{I}}_{{\text{p}}} \left( {{\text{nA}}} \right) = - 0.{8684} + {1}.{8}0{\text{4lgC }}({\text{nmol}}/{\text{L}}), \, ({\text{R}}^{{2}} = 0.{9928}) $$

As shown in Fig. 6B, the linear equation range from 1.0 to 10 nmol/L was:

$$ \Delta {\text{I}}_{{\text{p}}} \left( {{\text{nA}}} \right) = 0.{2261} + 0.{\text{2875logC }}\left( {{\text{nmol}}/{\text{L}}} \right), \, ({\text{R}}^{{2}} = 0.{9931}) $$

The limit of detection (LOD, S/N = 3) was 0.88 nmol/L. The results indicate that this MB-Apt-S/GE can achieve rapid and sensitive quantitative analysis of MAMP.

At low MAMP levels the local concentration at the electrode surface is rapidly recognized, resulting in a high sensitivity of the electrode response. At higher MAMP concentrations, the aptamer is combined with substrate for a longer period of time and the reaction proceeds over a larger time window. This, together with the possibility of fouling of the electrode surface by the reaction products, results in a lower slope. Also, it attains a saturation level at higher concentration. Thus, the sensor showed different linear correlations at different concentration ranges^35^.

### Interference, repeatability, stability and reproducibility studies

**Figure 7:**
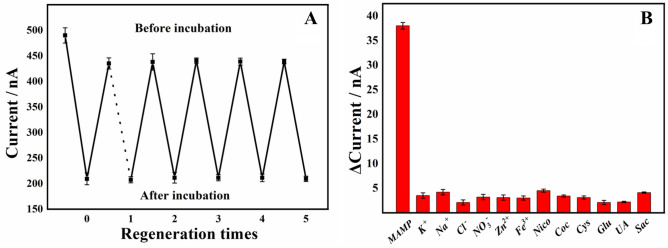
(**A**) Effect of number of activations on the peak current values of MB-Apt-S/GE before and after incubation in the sample solution. (**B**) Effect of different interferents on MB-Apt-S/GE current response values.

To assess the reproducibility of the modified electrode, the work electrode was used and the values of its current response signal in the supporting electrolyte before and after incubation were recorded. The results were shown in Fig. 7A. Although the MB signal of the modified electrode was attenuated after regeneration, it still showed a good electrochemical response to MAMP. The relative standard deviation (RSD) for six measurements was 3.9%, which demonstrated a good reproducibility of the modified electrode. The anti-interference performance was then investigated. Figure 7B shown the change in current response of the modified electrode to 200 nmol/L MAMP after the addition of different multiples of interferents. The results showed that 100 times of the inorganic salt ions of K^+^, Na^+^, Cl^−^, NO^3−^, Zn^2+^, Fe^3+^ and 50 times of the inorganic salt ions of nicotine (Nico), cocaine (Coc), uric acid (UA) cysteine (Cys), glucose (Glu) and sucrose (Sac) did not interfere significantly with the 200 nmol/L MAMP assay, demonstrating a good anti-interference ability of the modified electrode. After storing the modified electrode in 1 × PBS (pH = 7.4) buffer solution at 4 °C for 15 days, the current response value was 94.0% of the initial value, indicating that the electrode exhibited good stability. To evaluate the reproducibility of the modified electrode, two MB-Apt-S/GE were prepared under the same conditions using the same procedure, and three sets of parallel experiments were carried out, and the standard deviation of the current response was 2.3%, indicating that the modified electrode prepared by this method had good reproducibility.

### Detection of MAMP in real sample

To further evaluate the value of the prepared electrochemical aptamer sensor for practical applications, the MAMP content in real samples was measured using a standard curve method. As body fluid samples from drug users are difficult to obtain, urine and saliva from healthy humans were used to simulate the real sample environment and quantitative MAMP was added for the assay. Prior to spiking, saliva and urine were filtered and processed by using a 0.45 μm microporous membrane, diluted and added to the MAMP standard solution. The concentration of the substance to be measured in the sample solution before and after spiking was determined and the results were shown in Table 2. It can be seen that the MAMP recoveries ranged from 96.3 to 107% with an RSD less than 6.6%, indicating that the sensor is of good practical value.

**Table 2:** The analysis results of MAMP in saliva and urine (n = 5).

Samples	Added (nmol/L)	Found (nmol/L)	RSD (%, n = 5)	Recovery (%)
Saliva	0	0	–	–
	30.0	30.3	3.6	101
	40.0	38.6	3.3	96.7
	50.0	49.5	5.8	99.0
Urine	0	0	–	–
	30.0	31.9	4.5	107
	40.0	38.5	6.6	96.3
	50.0	48.1	5.9	96.3

Table 3 shows previously reported analytical determinations for MAMP, the detection range and LOD of this sensor is comparable to several other methods^33–39^. Those results indicated that this reagentless approach achieved nano-molar detection of MAMP in biological sample, which provided a rapid, sensitive and useful tool for the police to detect drugs on the spot quickly and conveniently.

**Table 3:** Comparison of analytical performance of different MAMP sensors.

Method	Modifier	Range (μM)	LOD (nM)	Samples	Refs.
Voltammetric (DPV)	PVDF-PEI/Anti-METH/GCE	2.0–50 ng/mL	0.007 ng/mL	Tear, sweat	^36^
Electrochemical-surface plasmon resonance (EC-SPR)	MDEA/DA/SPR	100–1,000,000	57, 59	10%FBA, urine	^37^
Voltammetric (FFT-SWV)	MIP/MWCNTs-CPE	0.01–100	0.83	Serum, urine	^38^
Voltammetric (SWV)	CeO2NP/rGO/GCE	25–166.6	8.7 × 10^3^	Plasma	^39^
Voltammetric (DPV)	1-Butyl-3-methylimidazolium bis (trifluoromethylsulfonyl) imide (BMIM TFSI)	5.0–1000 ng/mL	0.56 ng/mL	Saliva	^40^
Electrochemical aptamer-based sensor	Graphite SPE	0.02–20	20	Saliva, serum, urine	^24^
Voltammetric (SWV)	Graphite SPE	50–2500	16.66 × 10^3^	Seized sample	^41^
Voltammetric (DPV)	GE/Apta-4/METH	0.1–50 ng/mL	0.467 ng/mL	urine	^15^
Voltammetric (SWV), eATRP	DNA/sDNA	0.001–100	17fM	Serum, urine	^24^
Voltammetric (SWV)	Apt-I/GE	0.001–0.01, 0.01–0.4	0.88	Serum, urine	This work

## Conclusion

In this paper, an MB-Apt-S/GE electrochemical aptamer sensor was successfully prepared using meth-specific aptamer (Apt) as the recognition element. The performance of the modified electrode was investigated by CV, EIS and SWV. Under optimal experimental conditions, the modified electrode showed high selectivity to MAMP. The linear regression equation was: ΔI_p_ (nA) = -0.8684 + 1.804logc (nmol/L), (R^2^ = 0.9928), the linear equation range from 1.0 to 10 nmol/L was: ΔI_p_ (nA) = 0.2261 + 0.2875logC (nmol/L), (R^2^ = 0.9931) and the limit of detection (LOD, S/N = 3) was 0.88 nmol/L. The spiked recoveries of saliva and urine ranged from 96.3 to 107%. The results indicated that the sensor exhibited good electrochemical response to MAMP and can be used for the analysis of MAMP in human urine and saliva. The modified electrode was easy to be prepared and show excellent stability, anti-interference ability and reproducibility.

### Supplementary Information

Supplementary Information 1.

Supplementary Information 2.

Supplementary Information 3.

Supplementary Information 4.

Supplementary Information 5.

Supplementary Information 6.

Supplementary Information 7.

Supplementary Information 8.

Supplementary Information 9.

Supplementary Information 10.

Supplementary Information 11.

Supplementary Information 12.

Supplementary Information 13.

Supplementary Information 14.

Supplementary Information 15.

Supplementary Information 16.

## Data Availability

The datasets used and/or analysed during the current study available from the corresponding author on reasonable request.

## Abbreviations

MAMP: Methylamphetamine
Apt: Anti-meth thiolated aptamer
MB: Methylene blue
MB-Apt-S: MB modified thiolated aptamer
GE: Gold electrode
MB-Apt-S/GE: MB-Apt-S modified gold electrode
SWV: Square wave voltammetry
POCT: Point-of-care testing
NUODC: United Nations Office on Drugs and Crime
GC: Gas chromatography
HPLC: High-performance liquid chromatography
GC–MS: GC-mass spectrometry
ECL: Electrochemiluminescence
cDNA: Complementary DNA strand
LOD: The limit of detection
TCEP: Tris (2-carboxyethyl) phosphine hydrochloride
MCH: 6-Mercapto-1-hexanol
S–S bond: Disulfide bond
SWV: Square wave voltammetry
CV: Cyclic voltammetry
EIS: Electrochemical impedance spectroscopy
i_p blank_: The current response of the electrode incubated with the cDNA-only buffer solution
i_p MAMP_: The peak signal current with MAMP added to the cDNA solution
i_p initial_: Before the incubation, MB-Apt-S/GE was scanned using SWV obtained current value
RSD: Relative standard deviation
Nico: Nicotine
Coc: Cocaine
UA: Uric acid
Cys: Cysteine
Glu: Glucose
Sac: Sucrose
